# Supplementary material for: Predicting visual function by interpreting a neuronal wiring diagram
Source: Nature. 2024 Oct 2;634(8032):113–23. doi: 10.1038/s41586-024-07953-5 (PMC11446822; doi:10.1038/s41586-024-07953-5)
Supplement: Supplementary file 6 — CRF and ERF predictions for individual TmY4 and TmY9 cells. Analogous to Supplementary Data 3, but for TmY target types. Shown are the top four monosynaptic pathways, the strongest pathway passing through each of the top ten intermediary types (ranking from Extended Data Fig. 7), and the trisynaptic pathway Tm1–TmY–Dm3–TmY (see the section entitled Prediction of spatial normalization). [file 41586_2024_7953_MOESM6_ESM.zip › DataS4/TmY9qΓèÑ/720575940605522121.pdf]

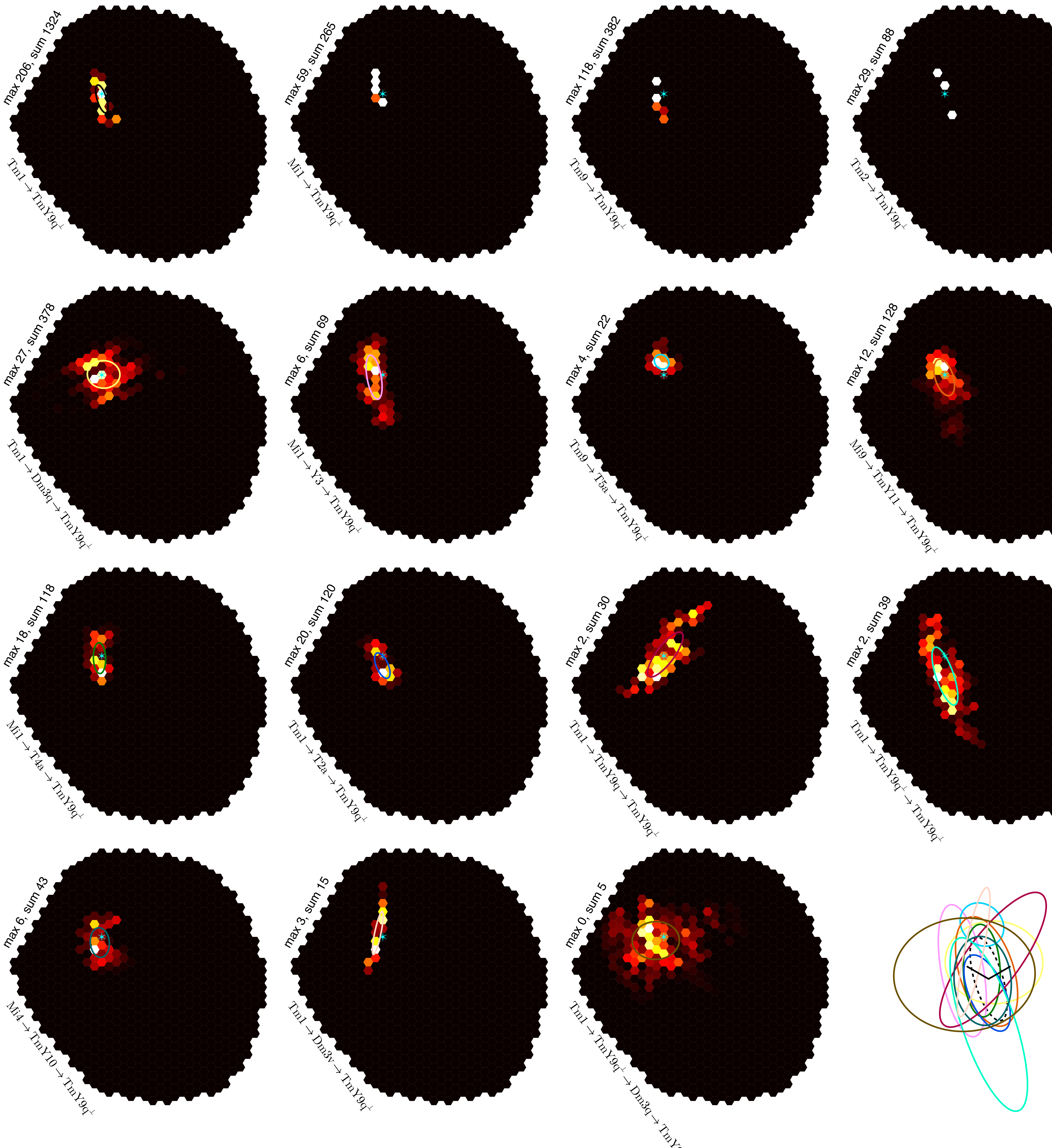

max 206, sum 1324
Tm1 → TmY9q⊥
max 59, sum 265
Mi1 → TmY9q⊥
max 118, sum 382
Tm9 → TmY9q⊥
max 29, sum 88
Tm2 → TmY9q⊥
max 27, sum 378
Tm1 → Dm3q → TmY9q⊥
max 6, sum 69
Mi1 → Y3 → TmY9q⊥
max 4, sum 22
Tm9 → T5a → TmY9q⊥
max 12, sum 128
Mi9 → TmY11 → TmY9q⊥
max 18, sum 118
Mi1 → T4a → TmY9q⊥
max 20, sum 120
Tm1 → T2a → TmY9q⊥
max 2, sum 30
Tm1 → TmY9q → TmY9q⊥
max 2, sum 39
Tm1 → TmY9q⊥ → TmY9q⊥
max 6, sum 43
Mi4 → TmY10 → TmY9q⊥
max 3, sum 15
Tm1 → Dm3v → TmY9q⊥
max 0, sum 5
Tm1 → TmY9q⊥ → Dm3q → TmY9q⊥
